# Molecular diagnosis of alveolar echinococcosis in patients based on frozen and formalin-fixed paraffin-embedded tissue samples

**DOI:** 10.1051/parasite/2022004

**Published:** 2022-02-02

**Authors:** Jenny Knapp, Séverine Lallemand, Franck Monnien, Sophie Felix, Séverine Valmary-Degano, Sandra Courquet, Florent Demonmerot, Bruno Heyd, Celia Turco, Alexandre Doussot, Lucie Bourgeois, Solange Bresson-Hadni, Carine Richou, Laurence Millon

**Affiliations:** 1 Department of Parasitology-Mycology, National Reference Centre for Echinococcoses, University Hospital of Besançon 25030 Besançon France; 2 UMR CNRS 6249 Laboratoire Chrono-environnement, Université Bourgogne-Franche-Comté 16 Route de Gray 25030 Besançon France; 3 Department of Pathology, University Hospital of Besançon 25030 Besançon France; 4 Department of Pathology, University Hospital of Grenoble-Alps 38043 Grenoble France; 5 Visceral, Digestive and Cancer Surgery, Hepatic Transplantation Unit, University Hospital of Besançon 25030 Besançon France; 6 Department of Digestive Surgery, Hepato-Biliary-Pancreatic and Liver Transplantation, AP-HP Pitié-Salpêtrière Hospital – Charles-Foix 75651 Paris France; 7 Department of Hepatology, University Hospital of Besançon 25030 Besançon France

**Keywords:** *Echinococcus multilocularis*, Molecular diagnosis, Fresh material, FFPE, End-point PCR, qPCR

## Abstract

Confirmed diagnosis of alveolar echinococcosis (AE) is based on pathological criteria and molecular evidence. This parasite-borne disease, caused by the cestode *Echinococcus multilocularis*, sparingly involves humans as a dead-end host. In humans, the parasite mainly colonizes the liver but can colonize any organ and cause atypical forms, often difficult to characterize clinically. Moreover, molecular methods may be suitable to make the diagnosis of AE in cases of atypical forms, extra-hepatic localizations, or immunosuppressed patients. The aim of this study was to determine the most relevant published PCR techniques, for diagnosis of AE in patients and adopt the best strategy for molecular diagnosis depending on the nature of the tested sample. In this study, we evaluated nine end-point PCR assays and one real-time PCR assay (qPCR), targeting mitochondrial genes, using a total of 89 frozen or formalin-fixed paraffin-embedded (FFPE) samples from either 48 AE or 9 cystic echinococcosis patients. Targeted fragment-genes ranged from 84 to 529 bp. Six PCR assays were able to amplify the DNA of 100% of the frozen AE-samples and for one PCR, 69.8% of the FFPE AE-samples. The 16S rrnL PCR (84 bp) was positive in PCR for 77% of the AE samples and in qPCR for 86.5%. The sensitivity of the PCR assays was higher for fresh samples and FFPE samples stored for less than 5 years. The qPCR assay further increased sensitivity for the tested samples, confirming the need for the development of an *Echinococcus* spp. qPCR to improve the molecular diagnosis of echinococcoses.

## Abstract (fr)

La confirmation diagnostique de l’échinococcose alvéolaire (EA) est basée sur des critères anatomo-pathologiques et moléculaires. Cette maladie d’origine parasitaire, causée par le cestode *Echinococcus multilocularis*, implique sporadiquement l’homme, impasse parasitaire. Chez l’homme, le parasite colonise principalement le foie mais peut coloniser tout organe et causer des formes atypiques, souvent difficiles à caractériser cliniquement. En outre, les méthodes moléculaires permettent de réaliser le diagnostic de l’EA dans les formes atypiques, les localisations extra-hépatiques ou chez les patients immunodéprimés. Le but de cette étude était de déterminer les techniques PCR publiées les plus pertinentes, pour le diagnostic de l’EA chez les patients et adopter la meilleure stratégie par diagnostic moléculaire en fonction de la nature de l’échantillon testé. Dans cette étude nous avons évalué neuf PCR en point-final et une PCR-temps-réel (qPCR), ciblant des gènes mitochondriaux, utilisant 89 échantillons congelés ou fixés en paraffine (FFPE) de patients EA (n = 48) ou présentant une échinococcose kystique (n = 9). Les fragments de gènes ciblés allaient de 84 à 529 pb. Six tests PCR ont permis d’amplifier l’ADN de 100 % des échantillons EA congelés, et pour une PCR, 69,8 % des échantillons EA-FFPE. La PCR 16S rrnL (84 pb) était positive en PCR pour 77 % des échantillons EA et en qPCR pour 86,5 %. La sensibilité des tests PCR était plus importante pour les échantillons congelés et les FFPE stockés moins de 5 ans. Le test qPCR a permis d’augmenter la sensibilité pour les échantillons testés, confirmant le besoin de développement d’une qPCR *Echinococcus* spp. pour améliorer le diagnostic moléculaire des échinococcoses.

## Introduction

**Figure 1:**
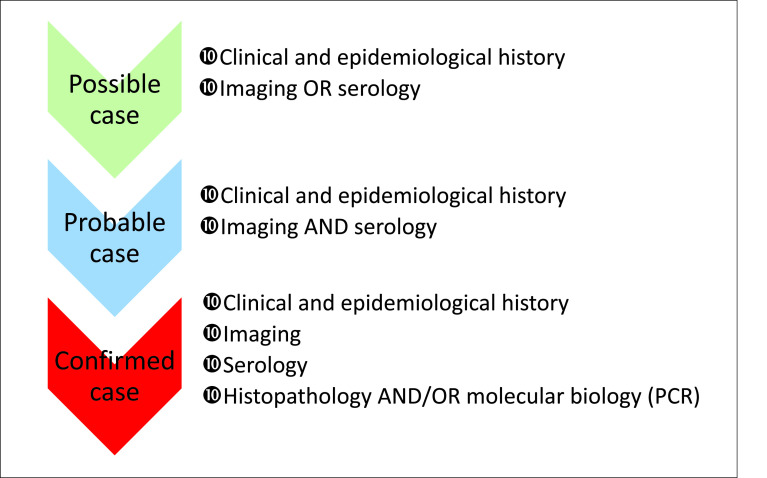
Classification criteria for alveolar echinococcosis cases, according to Brunetti et al. [7].

Alveolar echinococcosis (AE) is a serious and rare zoonosis, endemic to the northern hemisphere, with increasing incidence. The WHO has classified echinococcosis as one of the 17 neglected diseases to be controlled or eliminated by 2050. AE is caused by the cestode *Echinococcus multilocularis*, classified in the family Taeniidae, which contains three other genera: Taenia, Hydatigera, and Versteria [23]. The taxonomy of the genus *Echinococcus* has been established and includes 8 to 10 species, according to the latest publications, of which six have been described to be pathogenic for humans: *E. multilocularis*, *E. granulosus* sensu stricto, *E. canadensis*, *E. ortleppi*, *E. vogeli*, and *E. oligarthra* [32, 38, 40]. AE is caused by *E. multilocularis*, while cystic echinococcosis (CE) is caused by three species of the *E. granulosus* complex (*E. granulosus* sensu stricto, *E. canadensis* and *E. ortleppi*), and neotropical echinococcosis is caused by *E. vogeli* and *E. oligarthra*. The life cycle of *E. multilocularis* includes two mammals, canids (mainly red foxes *Vulpes vulpes* in Europe) as final hosts, and small mammals, such as rodents and, occasionally, humans as intermediate hosts [29, 36]. Humans are infected by the accidental ingestion of eggs released in canid feces. It is currently accepted that 1% of people who ingest the parasite actually develop the disease [3]. Initially, AE develops almost exclusively in the right lobe of the liver. The infection has a long course and mimics liver cancer, with characteristic granulomatous periparasitic and diffuse infiltration together with dense fibrosis [7, 37]. The treatment of choice is liver resection, whenever possible, with two years of albendazole (ABZ) treatment and, in other cases, continuous treatment with ABZ, but with a risk of many side effects, some of them being serious (e.g. elevation of serum transaminases, hepatic cytolysis, alopecia, teratogenicity observed in rats and rabbits) [7, 27]. Mortality is > 90% for patients not or inadequately treated [22]. Approximately 18,000 new cases appear each year in the world, including 16,400 in China and 1600 in Europe, and the average global burden is estimated to 666,434 disability-adjusted life years (DALYs) [33]. In France, the increasing number of cases appears to be linked to the increase in the number of immunosuppressed patients, with 19% of incidental AE cases being diagnosed in such patients from 2014 to 2018 [6, 8]. In an increasing number of cases (approximately 60%), the initial diagnosis is made by chance in asymptomatic patients or those with few symptoms, in most cases upon abdominal ultrasound examination [6]. The clinical diagnosis is confirmed by serology in 95% of cases with an enzyme-linked immune sorbent assay (ELISA) based on *E. multilocularis* antigens (Em2 and Em18) and immunoblotting tests (western blot) [41]. On histopathological examination, AE macroscopically shows an alveolar structure with numerous irregular millimetre-scale vesicles and microscopically, an intense periparasitic granulomatous reaction, with extensions to adjacent liver tissues, and a Periodic-Acid-Schiff (PAS)-positive laminated layer delimitating the vesicles, with protoscoleces absent in most cases [7, 24]. Molecular diagnosis is based on the detection of *E. multilocularis*-specific DNA sequences with polymerase chain reaction assays, with agarose gel visualization of the amplification products (hereinafter called end-point PCR or PCR), coupled with sequencing for confirmation. According to the recommendations of the WHO-Informal Working Group on Echinococcosis (WHO-IWGE) [7], probable cases are defined by clinical and epidemiological history, imaging findings, and serology positive for AE; only histopathology and molecular biology exams make a confirmed AE diagnosis (Fig. 1).

PCR can be carried out to confirm the AE diagnosis especially in patients with atypical lesions (25% of ultrasound results) [1], immunocompromized patients with negative serology, and patients with extrahepatic lesions (4% of cases) [18, 25]. PCR can be performed from frozen tissues (surgical specimens, fine needle biopsies), or fluids [6, 12, 28, 31]. Fine needle biopsies are now offered more frequently. Performing this technique under ABZ therapy is recommended to reduce the parasite dissemination risk during the procedure. PCR assays can also be performed on formalin-fixed paraffin-embedded (FFPE) tissues [30], in retrospective studies, or in case the diagnosis was missed in the first investigations. At the French National Reference Centre for Echinococcoses (NRC-E), 85% of samples received for molecular diagnosis from 2017 to 2019 were fresh samples (surgical specimens, fine needle biopsies, liquids) (*n* = 35) and 15% were FFPE samples (*n* = 6) (NRC-E data). Fresh samples are conserved frozen, at −20 °C at the NRC-E laboratory upon receipt. FFPE is a form of preservation for tissue specimens. After excision, the tissue is immersed in formalin to be fixed and then embedded in a paraffin wax block. Then, slices are cut with a microtome for further examination. FFPE is used for morphological diagnosis and immunohistochemical diagnosis in hematology, oncology, and immunology. Finally, FFPE tissues can also be a source of nucleic acids for molecular diagnosis. However, DNA degradation in FFPE samples is common and they often require more sensitive techniques. Successful molecular diagnosis relies on the choice of targets for the desired species, here *E. multilocularis*. A review of the literature led us to choose nine already published targets. Schneider et al. used a 252-bp target sequence (designed on the *nad1* gene) to detect *E. multilocularis* in FFPE tissues [30]. A species-specific target of 395-bp was chosen within the same gene by Trachsel et al. for the identification of taeniid eggs from carnivore feces by multiplex PCR associated with *E. granulosus* (PCR product of 117 bp) and *Taenia* spp. (PCR product of 267 bp) DNA detection [35]. Bowles et al. used a 446-bp sequence of an evolutionarily conserved region of the *cox1* gene, originally designed against *Fasciola hepatica*, to identify genetic variants within the genus *Echinococcus* sp. [4]. Stefanić et al. designed a PCR based on a 255-bp sequence of the mitochondrial 12S rRNA gene to detect *E. granulosus* sensu stricto in infected dogs [31]. Within the same target, a 200-bp fragment from the *E. multilocularis* 12S rRNA gene was used by Georges et al. for the diagnosis of AE in unusual localizations, such as bone [12]. In a previous study, we chose an 84-bp target located within the large ribosomal subunit gene (part of the 16S rrnL gene), initially designed to study environmental fecal contamination by *E. multilocularis* from red fox stools [19]. Roelfsema et al. designed primers to study a 268-bp target for the differential diagnosis of cestodes, which can differentiate all *Echinococcus* species after Sanger sequencing [28]. Finally, Bowles and McManus worked on two targets (183 and 529-bp) based on the *nad1* gene to differentiate species and genotypes of *Echinococcus* sp. [5].

This study is a comparison of nine previously published end-point PCR assays for the detection of Taeniidae in human samples and one qPCR assay. We aimed to determine the most relevant PCR assays for human diagnosis of AE in laboratories were both *E. multilocularis* and *E. granulosus* specimens can be addressed and adopt the best strategy for molecular diagnosis depending on the nature of the tested sample (frozen or FFPE specimens).

## Materials and methods

### Collection of *Echinococcus* spp. specimens and DNA extraction

The collection of 89 echinococcosis samples included 74 AE and 15 CE samples (Table 1), for differential diagnosis and to challenge the different PCR assays. The collection was composed of frozen specimens (25 samples frozen after surgery) and FFPE specimens (64 samples). The samples came from 57 echinococcosis patients with AE (27 men, 21 women, mean age at diagnosis 53.6 ± 17.0 years) or CE (6 men and 3 women, mean age at diagnosis 32.9 ± 16.2 years). The collection was thus composed of samples from AE and CE patients sent to the NRC-E from 1997 to 2019 (Table S1) for molecular diagnosis based on the 12S target [28] or the pathology laboratory for histological diagnosis (University Hospital Center of Besançon, France) (Table S1). The echinococcosis diagnosis was made first on imaging techniques and confirmed on histopathological criteria and/or molecular biology. The year of surgery or paraffin inclusion date was available for all the patients included in the present study. The nature of the samples studied is presented in Table 2.

**Table 1:** Collection of alveolar and cystic echinococcosis lesions stored frozen or as formalin-fixed paraffin-embedded (FFPE) samples, indicating the number of samples (number of patients).

	AE	CE	Total
Frozen specimen	20 (18)	5 (5)	25 (23)
FFPE	54 (30)	10 (5)	64 (35)
Total	74 (48)	15 (9)	89 (57)

**Table 2:** Locations of fresh and FFPE samples for alveolar and cystic echinococcosis lesions. ND for no data.

Nature of samples	FFPE		Total FFPE	Native		Total native	Total
Location	AE	CE		AE	CE		
Liver	27	2	29	16	0	16	45
Kidney	1	1	2	1	0	1	3
Hydatid cyst	0	0	0	0	3	3	3
Brain	0	0	0	1	0	1	1
Bone	0	1	1	0	0	0	1
Lung	0	0	0	1	0	1	1
Liquid from bone lesion	0	0	0	1	0	1	1
Other tissue	2	0	2	0	1	1	3
NA	0	1	1	0	1	1	2
Total	30	5	35*	20	5	25	60

*29 FFPE samples were extracted twice, 6 samples once.

The collection included 25 frozen samples, placed at −20 °C upon receipt and processed between April 2007 and January 2020 (20 samples from 18 patients diagnosed with AE and 5 samples from 5 CE patients, Table 2 and Table S1, initially confirmed by sequencing the 12S PCR products [28]). DNA was purified and re-extracted from tissues for the present study, using a High Pure PCR Template Preparation kit (Roche Diagnostics, Mannheim, Germany), on square millimetre pieces of the surgical sample, following the manufacturer’s recommendations, from January to February 2020. DNA was eluted in a volume of 200 μL of the provided buffer and stored at −20 °C until use.

The collection also included 64 FFPE specimens (54 samples from 30 patients histologically diagnosed with AE and 10 samples from 5 patients histologically diagnosed with CE, Table 2 and Table S1) to assess the feasibility of molecular retrospective studies on the diagnosis of echinococcosis. Samples were received by the pathology laboratory between 1997 and 2018. The duration of tissue formalin-fixation (10% formalin, pH = 7) varied from 24 to 72 h and the dehydration and impregnation cycles were processed using a Tissue-Tek VIP 6 device (Sakura Finetek, Tokyo, Japan). The mean time between surgery and paraffin inclusion was 2.9 ± 1.5 days and the mean time between paraffin inclusion and DNA extraction was 10.1 ± 7.1 years. Between three and ten 8-μm-thick paraffin shavings, depending on the sample, were obtained using a Microm HM330 microtome (Microm Microtech, Heidelberg, Germany). The shavings were de-paraffined within a week after obtaining the paraffin slides. Briefly, 1 mL xylene was applied to the sample, the sample mixed by vortexing for 1 min, and then centrifuged at 20,000 ×*g* for 2 min. Xylene was removed and 1 mL absolute ethanol applied, the sample washed by vortexing, and a new centrifugation performed (same conditions) before removing the ethanol and drying the pellet at 37 °C for 30 to 45 min. DNA was purified and extracted immediately after de-paraffining the FFPE shavings using a QIAamp DNA FFPE Tissue kit (Qiagen, Hilden, Germany), following the manufacturer’s recommendations. Two DNA extractions were performed for FFPE samples for 29 of the 35 patients (24 AE and 5 CE patients) in elution volumes of 50 μL (from April to September 2018) and 200 μL (from July to November 2019) of the provided buffer. Five of the remaining FFPE samples were eluted in 50 μL and one in 200 μL (from August 2017 to August 2018). DNA samples were stored at −20 °C until use. The DNA concentration was determined from 2 μL using a nanophotometer apparatus (Implen, Munich, Germany). The 15 samples of *E. granulosus* allowed us to define the primer specificity for the differential diagnosis between the two echinococcosis diseases. Five DNA extracts from other requests to search for the presence of *Echinococcus* DNA (three hepatic abscesses, one mediastinal cyst, and one healthy brain sample from an AE patient), received by the NRC-E and found to be negative for the parasite DNA (12S PCR) were used in each PCR as controls.

### Molecular diagnosis

**Figure 2:**
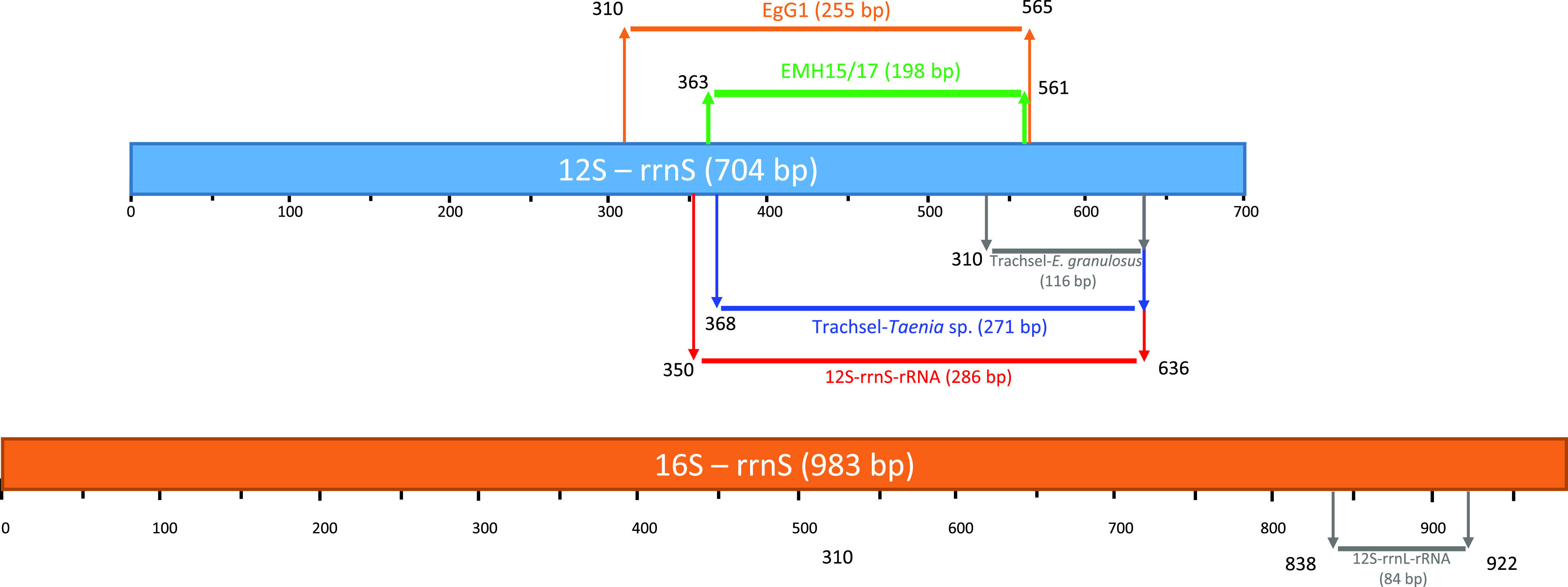
Diagram of the 12S RNA gene with location of the various targets used to amplify fragments from 116 to 286-bp by end-point PCR techniques.

Nine different end-point PCR assays and one qPCR assay based on the literature [4, 12, 19, 28, 30, 31, 35] were tested using the samples, targeting four mitochondrial gene fragments from 84 to 529 bp (Table 3). For the 12S-rrnS gene, the locations of the various targets used within the gene are shown in Figure 2. All but the multiplex PCR were carried out in a final volume of 20 μL, containing 10 μL of the 2× AmpliTaq Gold 360 master mix, with hot start AmpliTaq Gold 360 polymerase (Thermo Fisher Scientifics, Vilnius, Lithuania), 0.5 μM of the forward and reverse primers, and 1 μL genomic DNA. The multiplex PCR B « Trachsel » was conducted in a final volume of 25 μL, containing 12.5 μL master mix (Qiagen multiplex PCR kit, Qiagen, Hilden, Germany), 2.5 μL primer mix (primers Cest1, Cest2, Cest3, and Cest4 at 2 μM and primer Cest5 at 16 μM), 9 μL water, and 1 μL DNA. Samples eluted in a volume of 50 μL elution buffer were used at 50 ng/μL or less and samples eluted in 200 μL were used without dilution (Table S1). PCR conditions were applied as recommended in the literature (Table 3) using a Biometra T3 thermocycler (Whatman Biometra, Göttingen, Germany). For each PCR set, controls were included: positive controls used at 1 ng/μL with *E. multilocularis* DNA extracted from adult worms isolated from a red fox, *E. granulosus* sensu stricto DNA extracted from protoscoleces isolated from hydatid fluid from patients, and *Taenia solium* from cysticercus for PCR B, and negative controls with a panel of five DNA extracts from other requests. Amplicons were visualized by electrophoresis, in which 8 μL of each PCR product was loaded onto 1.5% (w/v) agarose gels first stained with 10% SYBR Safe DNA Gel Stain (Invitrogen, Carlsbad, CA, USA) in 1× tris-acetate EDTA solution, run for 30 min at 100 V, and viewed under UV light using a Gel Logic 100 Imaging System and the associated software Scientific Imaging System v3.6.1 (Kodak, New Haven, CT, USA). The position and number of bands were noted for each PCR.

**Table 3:** Molecular targets tested, PCR conditions, and references.

PCR name (target gene)	Primer name	Primer sequence (5′ – 3′)	Size (bp)	Originally designed on	Species targeted and specific detection	Ref.	Cycling conditions
A-16S*	Em rrn-F	CTGTGATCTTGGTGTAGTAGTTGAGATTT	84	*Echinococcus multilocularis*	*Echinococcus multilocularis*	[19]	10 min/95 °C, 40 cycles: 30 s/94 °C, 30 s/60 °C, 10 s/72 °C
(16S-*rrn*L)	Em rrn-R	GGCTTACGCCGGTCTTAACTC					
	Hydrolysis probe	FAM-TGGTCTGTTCGACCTTTTTAGCCTCCAT-TAMRA					(RT-cycles) 2 min/50 °C, 10 min/95 °C, 40 cycles: 75 s/95 °C, 1 min/60 °C
B1-2-Multiplex TRACHSEL	Cest1	TGCTGATTTGTTAAAGTTAGTGATC	395	*Echinococcus multilocularis*	*Echinococcus multilocularis*	[35]	(Multiplex PCR) 40 cycles: 30 s/94 °C, 90 s/58 °C, 10 s/72 °C
	Cest2	CATAAATCAATGGAAACAACAACAAG					
(*nad1* and	Cest4	GTTTTTGTGTGTTACATTAATAAGGGTG	117	*Echinococcus granulosus*	*Echinococcus granulosus*		
12S-*rrn*S)	Cest5	GCGGTGTGTACMTGAGCTAAAC			Sensu stricto		
	Cest3	YGAYTCTTTTTAGGGGAAGGTGTG	267	*Taenia* spp.	*Taenia* spp.		
	Cest5	GCGGTGTGTACMTGAGCTAAAC					
C-JB11.5	ND1 JB11.5	TTATGGTAGATATTATAG	183	*Echinococcus granulosus*	*Echinococcus* spp., 1	[5]	40 cycles: 30 s/94 °C, 30 s/50 °C, 30 s/72 °C
(*nad1*)	ND1 JB12.5	CACACACATAAAACAAGC			mutation between G6/G7		
D-EMH15 (12S-*rrn*S)	EM-H15	CCATATTACAACAATATTCCTATC	200	*Echinococcus multilocularis*	*Echinococcus multilocularis*	[12]	40 cycles: 30 s/95 °C, 30 s/55 °C, 30 s/72 °C
	EM-H17	GTGAGTGATTCTTGTTAGGGGAAG					
E-EM29	EM29	GATTTGCTGATTTGTTAAAGTTAGTGATC	252	*Echinococcus multilocularis*	*Echinococcus multilocularis*	[30]	45 cycles: 30 s/95 °C, 45 s/56 °C, 60 s/72 °C
(nad1)	EM281	AGAACTTAAAAACGAATATTTATTGTAACT					
F-EG1	Eg1f	CATTAATGTATTTTGTAAAGTTG	255	*Echinococcus granulosus*	*Echinococcus granulosus*	[31]	40 cycles 30 s/94 °C, 30 s/53 °C, 45 s/72 °C
(12S-*rrn*S)	Eg1r	CACATCATCTTACAATAACACC			Sensu stricto		
G-12S	12S-Echino-F	AAAKGGTTTGGCAGTGAGYGA	268	*Echinococcus* spp.	*Echinococcus* spp.	[28]	40 cycles: 30 s/94 °C, 30 s/55 °C, 1 min/72 °C
(12S-*rrn*S)	12S-Echino-R (Cest5)	GCGGTGTGTACCTGAGCTAAAC					
H-COX1	CO1-F	TTTTTTGGGCATCCTGAGGTTTAT	446	*Fasciola hepatica*	*Echinococcus* spp., 1	[4]	30 cycles: 30 s/94 °C, 40 s/52 °C, 45 s/72 °C
(*cox1*)	CO1-R	TAAAGAAAGAACATAATGAAAATG			mutation between G6/G7		
I-JB11	ND1 JB11	AGATTCGTAAGGGGCCTAATA	529	*Fasciola hepatica*	*Echinococcus* spp., 4	[5]	40 cycles: 30 s/94 °C, 30 s/50 °C, 60 s/72 °C
(*nad1*)	ND1 JB12	ACCACTAACTAATTCACTTTC			mutations between G6/G7		
J-Alea	Alea-F	CCTAAAAATGTCTATGATTGGTCCACTA	167	Random nucleic sequence	Alea plasmid	[20]	(qPCR) 2 min/50 °C, 10 min/95 °C, 40 cycles: 75 s/95 °C, 1 min/60 °C
	Alea-R	GGGAGTACCTTGCCATACAAAATT					
	Alea-probe	VIC-TTAAATCAACTCCTAAATCCGCGCGATAGG-TAMRA					

* Target used for real-time quantitative PCR.

PCR A, designed from the mitochondrial target 16S-rrnL rRNA (84 bp between the positions 839 and 922 bp of the gene) was additionally tested by qPCR, as described by Knapp et al. in 2014 [19]. Briefly, the qPCR was run by using 2× TaqMan Gene Expression master mix (Life Technologies, Foster City, CA, USA), on an Applied Biosystems 7500 Fast Real-Time PCR System machine (Life Technologies) for 45 cycles as described by the authors [20] (Table 3). Quantitative results were expressed by determining the quantification cycle (Cq), the cycle at which fluorescence of the isolate became significantly different from the baseline signal. Each sample was amplified in duplicate and the average Cq number calculated. The presence of PCR inhibitors was controlled by the internal control Alea PCR, based on random DNA sequence amplification, in a PCR 16S/Alea duplex [20]. The Cq value for the detection of the PCR inhibitor was > 36 cycles as determined and recommended by the author [20]. The PCR results were analyzed using Applied Biosystems 7500 Real-Time PCR software v2.3 (Life Technologies).

### Data analysis

Performance in terms of sensitivity (Se) and specificity (Sp) for each specific PCR technique (A, B1-2, D and E) was assessed by comparing the results obtained among the *E. multilocularis* and *E. granulosus* specimens tested. Percentages were analyzed using Chi-square tests and the means using Student *t*-tests. *P*-values < 0.05 were considered significant. The year of surgery or paraffin inclusion being available, as the DNA extraction date, the relationship for the time interval between sampling and DNA extraction and the number of positive PCR assays was tested. Statistical analyses were performed using R (v4.0.3) [26] and the package Rmisc [15] for the 95% confidence interval (CI).

## Results

### End-point PCR

For all positive controls, obtained from pure materials (*E. multilocularis*, *E. granulosus* and *T. solium*), a band on the agarose gel was visualized at the expected size and the expected species for each specific PCR with a DNA concentration at 1 ng/μL, except for the NAD1 JB11.5 PCR (C) and the COX1 PCR (H), with a DNA concentration increased at 5 ng/μL, because of weak bands observed.

**Figure 3:**
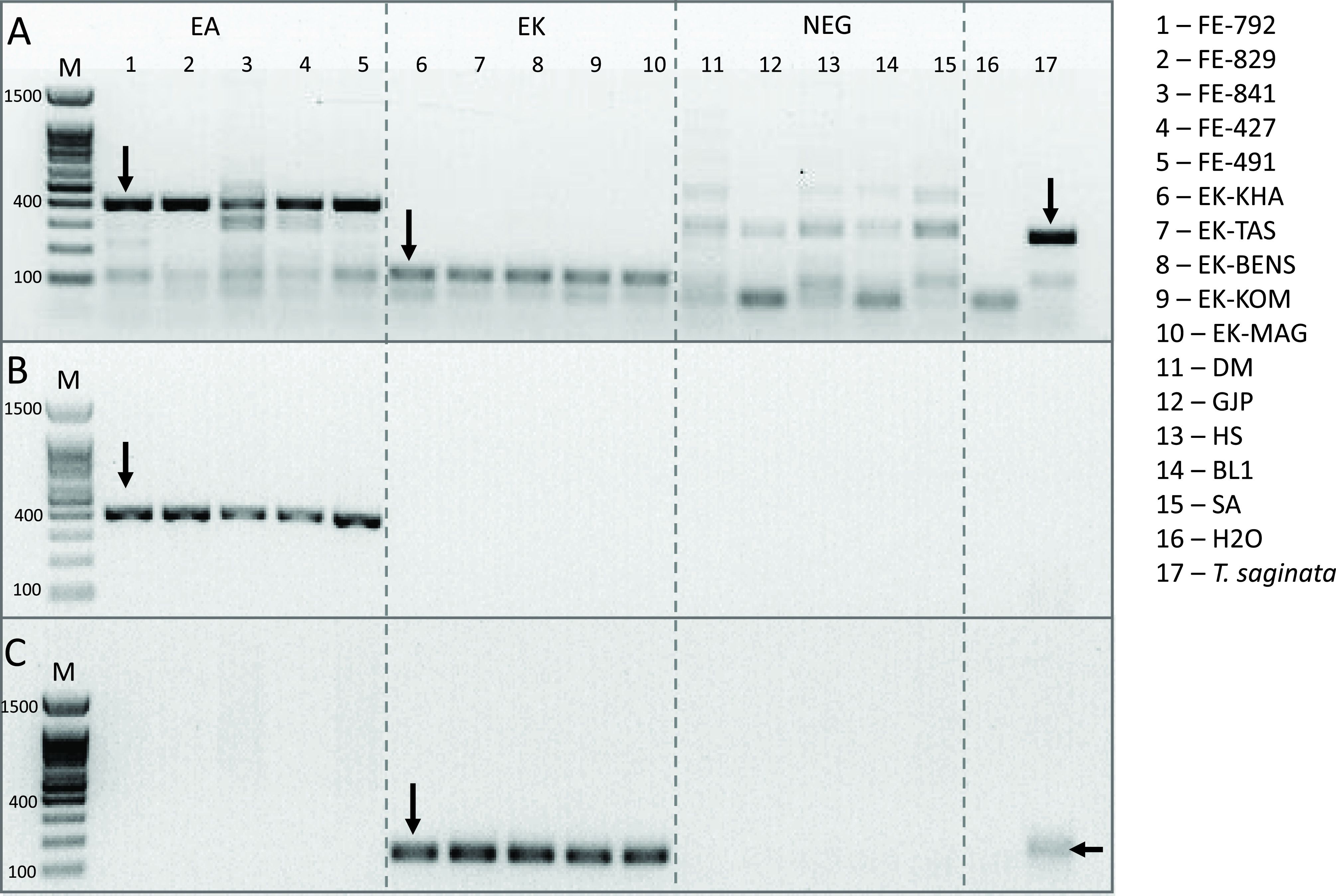
PCR products separated by electrophoresis on a 1.5% agarose gel showing multiband patterns for five AE, five CE, and five control patients (other requests). PCR assay B1-2 [35]. (A) Results for the multiplex PCR. (B) Results for the simplex PCR for *E. multilocularis* amplification. (C) Results for the simplex PCR for *E. granulosus* amplification.

**Figure 4:**
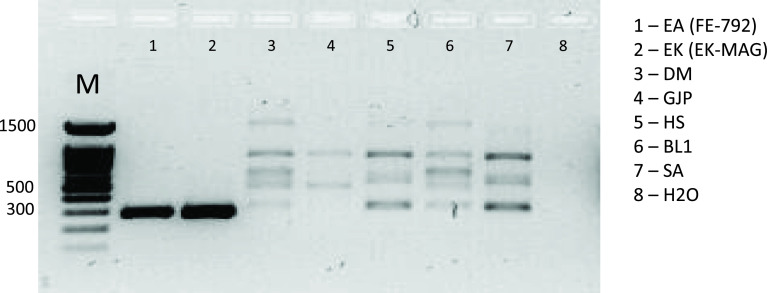
PCR assay G (12S) for negative control and positive AE and CE samples. Lane M: size marker.

PCRs on fresh samples were performed after DNA extraction after a mean time interval of 23.8 days [95% confidence interval (CI): 15.04; 32.56]. PCRs on FFPE samples were performed after DNA extraction after a mean time interval of 11.42 days [95% CI: −0.61; 23.46]. The results for the PCRs were controlled by checking for the presence or absence of a single band of the expected size for each technique on the agarose gels (Table 4 and Table S1). Multiple bands were observed for two assays (Trachsel PCR (B1-2) and 12S PCR (G)) and a single band for the other assays for positive tests. The DNA extracts from other requests were negative for all the PCR assays, but the multiplex PCR B1-2 and PCR G, where nonspecific bands were observed (Figs. 3A and 4). For these negative control samples, the PCR G products were sequenced (Sanger method) and compared to the GenBank database. The requested sequences were all similar to the reference sequence AC187125.3 (*Pan troglodytes*).

**Table 4:** PCR results according to *Echinococcus* species and sample type. N: number of samples tested, n: number of positive samples, *53 AE FFPE samples tested instead of 54, Se: sensitivity, and Sp: specificity, Em: *E. multilocularis*, Eg: *E. granulosus*, E spp.: *Echinococcus* spp.

Target gene		16S-*rrn*L-rRNA	12S-*rrn*S rRNA	*nad1*	12S-*rrn*S rRNA	*nad1*	12S-*rrn*S rRNA	12S-*rrn*S rRNA	*nad1*	*cox1*	*nad1*
PCR name		A	B1	C	D	E	F	G	B2	H	I
		16S	Trachsel EG	JB11.5	EMH15	EM29	EG1	12S	Trachsel EM	COX1	JB11
Target species		Em	Eg	E spp.	Em	Em	Eg	E spp.	Em	E spp.	E spp.
PCR product size (bp)		84	117	183	200	252	255	268	395	446	529
AE frozen (*N* = 20)	Se (n)	100% (20)	/	95.0% (19)	100% (20)	100% (20)	0% (0)	100% (20)	100% (20)	60.0% (12)	100% (20)
CE frozen (*N* = 5)	Se (n)	0% (0)	100% (5)	100% (5)	0% (0)	0% (0)	100% (5)	100% (5)	0% (0)	80% (4)	0% (0)
	Sp for AE diagnosis	100%	/	–	100%	100%	100%	–	100%	–	–
AE FFPE (*N* = 54)	Se (n)	69.8% (37*)	/	1.9% (1)	33.3% (18)	33.3% (18)	0% (0)	31.5% (17)	18.5% (10)	3.7% (2)	13.0% (7)
CE FFPE (*N* = 10)	Se (n)	0% (0)	20% (2)	0% (0)	0% (0)	0% (0)	0% (0)	0% (0)	0% (0)	0% (0)	0% (0)
	Sp for AE diagnosis	100%	/	–	100%	100%	/	–	100%	–	–

The end-point PCR A assay (16S, 84 bp) amplified the DNA of the most AE specimens, with 77.0% (57/74) positive results (100% for frozen samples and 69.8% for FFPE samples) (Table 4 and Table S1). The end-point PCR D and E assays (EMH15/H17 and EM29/281) both amplified 51.4% of the AE samples (38/74 AE samples, 100% for frozen samples and 33.3% for FFPE samples) and were both positive for the same *E. multilocularis* samples. For PCR E, the DNA extraction of the AE-FFPE samples found to be negative was tested using 1:10 and 1:50 dilutions of the 200 μL elution volume, as recommended by the author [30]. One sample (FE-693) was positive at a 1:10 dilution. The second FFPE-sample for this patient (50 μL elution volume) was initially positive without dilution.

Five specific PCR assays (PCR A, B2, D, E, and F) were tested. Four (PCR A, B2, D, and E for 16S) were 100% specific for *E. multilocularis* and PCR F (EG1) was 100% specific for *E. granulosus*, but only frozen DNA samples were amplified. All specific PCR assays showed 100% sensitivity and specificity for the selected targets for the AE and CE frozen samples. For the FFPE samples, the sensitivity ranged from 18.5 to 69.8% for the AE samples and from 0 to 20% for the CE samples, with 100% specificity.

Twenty-two AE samples (all FFPE samples) gave a positive result with only one PCR assay: 19/22 (86.4%) with only the specific PCR A and 3/22 (13.6%) with only the pan-*Echinococcus* specific PCR G. In total, 22 samples (24.7%, 22/89 samples) were negative for all nine PCR assays, all FFPE samples. The mean time interval between surgery and paraffin inclusion and that between paraffin inclusion and DNA extraction did not differ between samples negative for all the PCR assays and those positive for at least one (*p* = 0.095).

Two assays (PCR B and G) showed a multiband profile on the electrophoresis gels. For PCR B1, the specific 117-bp-band for *E. granulosus* appeared for all frozen CE samples, as well as for AE samples, along with additional bands between 200 and 400 bp (Fig. 3A). The specific 395-bp-band for *E. multilocularis* appeared for AE samples only (Fig. 3B). Weak bands from 300 to 500 bp were observed for the negative control panel. PCR B1-2 was performed in simplex and unique bands at 395 bp and 117 bp were observed for the AE and CE samples, respectively (Fig. 3C). The PCR performed with the pure positive controls presented each a single band.

For differential diagnosis on *E. granulosus* sensu stricto specimens, the sensitivity was 100% on fresh material for PCR B1 (Trachsel EG) and F (EG1). The pan-*Echinococcus* PCR G (12S) provided a sensitivity of 100% for fresh material but was not contributive for CE-FFPE samples.

### E. *multilocularis*-specific qPCR

The *E. multilocularis* positive control tested in duplicate with the qPCR 16S with a DNA concentration at 1 ng/μL had a mean Cq of 25.2, the *E. granulosus* control sample was negative (Cq > 45). For the AE samples tested, the qPCR was positive for 86.5% of the specimens (64/74), 100% for all AE-frozen samples (20/20, mean Cq = 27.1 [95% CI: 25.6; 28.6]), and 81.5% for AE-FFPE samples (44/54, mean Cq = 33.1 [95% CI: 31.4; 34.7]). One CE patient on FFPE-sample (patient No 21, Table S1) was positive for one DNA extract (50 μL) once in the PCR duplicate (Cq = 38.11). Among the 22 samples negative for all nine end-point PCR assays, eight AE samples (6 patients) were positive with the qPCR. For all PCR assays combined, 14 samples remained negative (7 AE samples from 4 patients and 7 CE samples from 4 patients). No PCR inhibitors were detected for the entire collection of samples based on the internal control qPCR Alea results.

### Number of effective PCR assays and storage time

**Figure 5:**
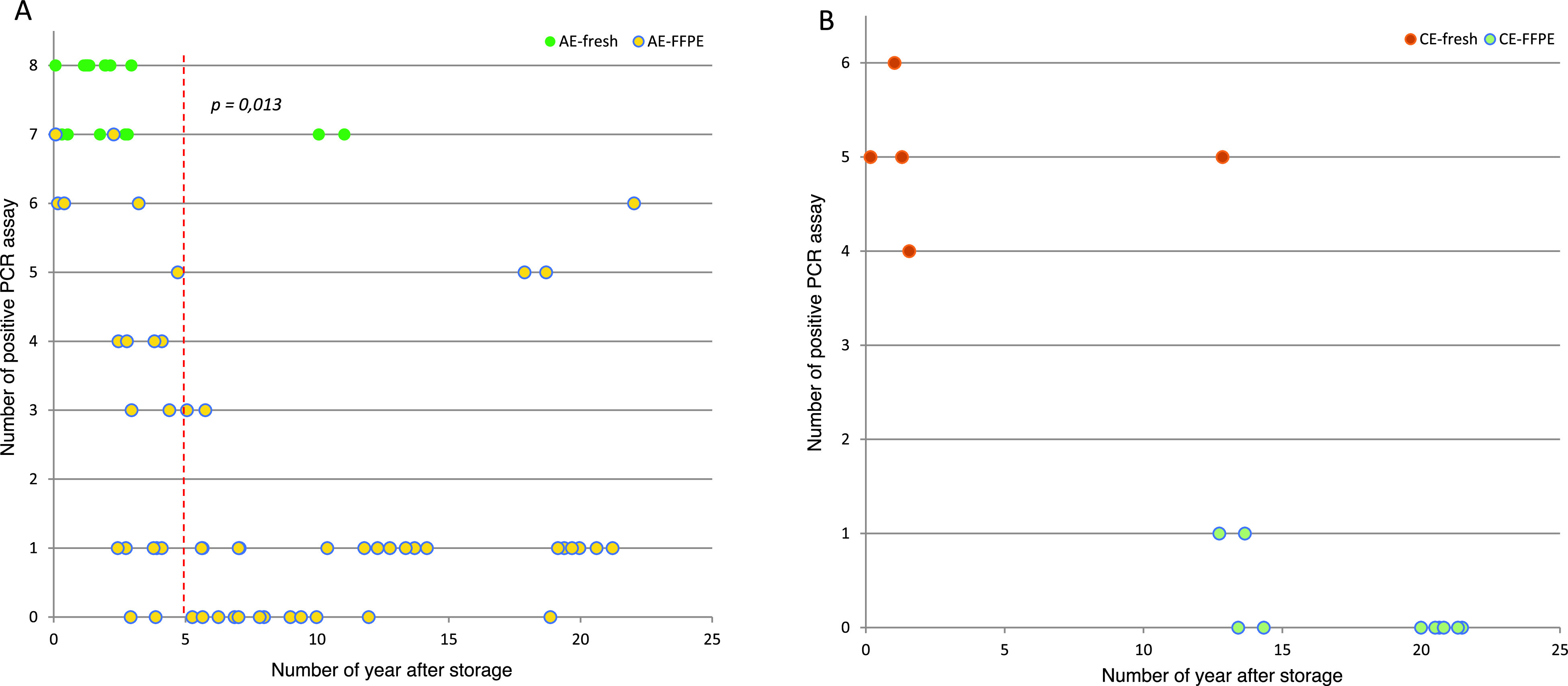
Number of positive PCR assays according to the time between starting point of the storage and DNA extraction and the type of sampling (fresh versus FFPE) and *Echinococcus* species. (A) The green and yellow dots represent fresh and FFPE AE samples, respectively. The dashed red line indicates for the FFPE samples when a significant difference in terms of positive PCR assays is found between the samples stored less than 5 years and more than 5 years. (B) The orange and light blue dots represent fresh and FFPE CE samples, respectively.

The relationship for the time interval between sampling and DNA extraction and the number of positive PCR assays is presented in Figures 5A and 5B. The mean time interval for frozen samples was 2.4 years [95% CI: 1.0; 3.7] for AE and 3.4 years [95% CI: −3.2; 10.0] for CE. For the FFPE samples, the mean time interval was 8.6 years [95% CI: 6.9; 10.4] for AE and 17.9 years [95% CI: 15.2; 20.6] for CE. For FFPE, the number of samples with at least one positive PCR assay was significantly higher for samples stored less than five years before DNA extraction (Chi-2 test, *p* = 0.013). However, correct amplification was observed for old samples as well, as for three AE samples (samples FE-168B and FE-270A and B, Table S1) that had been stored for over 17 years and with which at least five PCR assays were positive. The mean time interval for samples negative for all assays was 8.1 years [95% CI: 5.8; 10.4] for AE and 19.1 years [95% CI: 16.3; 21.8] for CE.

## Discussion

Accurate diagnosis of AE is necessary for proper disease management. Pathology and molecular diagnosis are currently the two methods used to confirm echinococcosis before a surgical procedure, performed on fine needle biopsies, under ABZ therapy [6] or after a surgical procedure on tissues. PCR techniques are particularly important in immunosuppressed patients with a median time to diagnosis of approximately five months due to unusual presentation, negative serology and/or pathological exam, metastasis, lymphoma-like lesions, or concomitant infections [8]. PCR assays allow for rapid diagnosis based on biopsied lesions and avoid potentially harmful treatment to the patients. The efficiency of PCR has been highlighted in cases of unusual localization (e.g. spleen, peritoneum, lungs, vertebra, brain, kidneys, heart), which represent 2.3% of primary lesions [12, 18].

In the present study, 89 samples from 57 echinococcosis patients were tested with nine end-point PCR assays and one qPCR assay to characterize the best protocol for molecular diagnosis, adapted to the nature of the sample. PCR sensitivity was better for frozen than FFPE samples. Two issues concerning storage must be considered, first the storage conditions of the tissue containing the DNA, and second, the storage conditions of the isolated DNA itself after purification. The amount of DNA extracted from frozen tissues using commercial kits has been found to be higher than that for FFPE samples for a collection of non-tumoral samples, even for recent FFPE preparations [11]. Moreover, the success of PCR was higher from frozen tissues than from at least one-year-old FFPE samples. For FFPE samples, the amount of time between fixation and DNA extraction is critical for DNA quality, as are the material used and the type of tissue. Moreover, variations in the different steps of FFPE preparation, especially formalin fixation, play a role in DNA fragmentation and PCR inhibition. Variations in DNA extraction steps for FFPE samples can also influence the quality of the DNA obtained [21, 30]. For DNA storage, two strategies are commonly used: storage at room temperature on a solid dry matrix or storage at cold temperatures, from 4 °C to −196 °C [2]. For dry storage, changes in the amount of moisture can damage the DNA because of hydrolysis. Environmentally derived DNases can also lead to DNA degradation. Conventionally, colder temperatures are recommended for DNA storage, leading to less DNA fragmentation. However, storage at 4 °C for short-term analyses can be a good compromise to avoid multiple freeze-thaw cycles.

We found the PCR A target originally designed for environmental contamination screening and the detection of *E. multilocularis* in red fox stools by qPCR [19] to be positive for 100% of the frozen AE samples and 69.8% of the FFPE-AE samples. This target presented the best combined performance in terms of sensitivity and specificity for the entire set of specimens. This short 84-bp target has an advantage as a PCR target because small fragments have a higher probability of being detected when DNA undergoes degradation, and it is highly specific for *E. multilocularis*. However, its short length makes it difficult to sequence the PCR product for formal species confirmation, but ligation of the PCR products into a plasmid vector and sequencing could solve the problem. The PCR A target is therefore useful as a rapid screening method either in end-point PCR A or qPCR coupled to the Alea-qPCR internal control to check for the presence of inhibitors, and confirming the need for the development of a multiplex *Echinococcus* spp. qPCR to improve the molecular diagnosis of echinococcoses.

PCR D and E also gave good results in terms of specificity, but showed lower performance in terms of sensitivity for FFPE samples. On the other hand, they have the advantage giving long PCR products for formal identification by sequencing. PCR E was initially designed for FFPE specimens. The specificity of the PCR was confirmed in the present study, but was not as high as previously described. In a study of Schneider et al., the authors amplified samples stored in paraffin for up to 30 years and obtained better results for inhibited samples after dilution. In the present study, the frozen samples were successfully amplified, but only one third of the FFPE samples were correctly amplified and one sample only became positive after a second PCR performed on a 1:10 dilution of the DNA. Nonetheless, amplification may be difficult with fragmented samples for a target of 252 bp, which could be the case here.

In cases of *Echinococcus*-negative samples, multiband patterns appeared on the agarose gels, but the sequenced PCR products were the result of human DNA amplification, certainly due to cross-reaction with the host-DNA during PCR. This phenomenon was previously described by Grimm et al. and attributed to a large amount of host cell DNA present in the DNA extract [13]. Moreover, from our study, the use of pure positive controls, isolated from distinct parasite specimens from the host (adult worms and protoscoleces) is confirmed for proper interpretation of results. PCR B is a multiplex PCR designed to target *E. multilocularis*, *E. granulosus*, and *Taenia* spp. in a single reaction mixture [35]. The 117-bp band detected on agarose gels, apparently specific for *E. granulosus*, could be visualized for all frozen CE samples, but was also detected for some AE samples. In simplex PCR, the specific bands were observed for the *E. granulosus* and *E. multilocularis* samples. The multiband pattern observed is likely due to the primer mixture used. Simplex PCR should be conducted secondarily to confirm the *Echinococcus* species. Furthermore, Trachsel et al. detected *E. vogeli* DNA with this target when screening for *E. granulosus* DNA, highlighting its lack of specificity [35]. Nevertheless, this assay is still a robust helpful PCR and the only multiplex PCR technique described in the literature for Taeniidae DNA detection.

Among the 89 samples of the study, all frozen tissues (*n* = 25) were positive for at least four PCR assays. However, the frozen specimens were relatively more recent than the FFPE samples and older frozen samples for a complete comparison were lacking, especially in quantification by qPCR among fresh and FFPE samples, as observed in the literature [14]. None of the nine PCR assays were positive for 22 FFPE samples. However, eight negative samples were positive with the qPCR. Finally, in total, 14 samples were negative with all assays, all being FFPE samples that were embedded in paraffin more than six years before. Given the mean time interval between storage and DNA extraction, greater DNA degradation is likely the cause of the absence of amplification. Nucleic acids extracted from paraffin blocks are highly degraded in blocks stored more than 4 years, because DNA stability in conserved FFPE material decreases [13], with residual fragments of mostly < 300 bp, formation of DNA-protein crosslinks, increasing the sensitivity of DNA to mechanical stress and decrease the accessibility for enzymes. When formalin is oxidized to formic acid, DNA depurination and DNA strand breaks can be observed [9]. This must be considered, especially for retrospective studies. As described in the literature, PCR is more sensitive for FFPE samples stored for less than five years [14, 16, 30], and this was emphasized in our study. This can be explained by the continual degradation of DNA by formalin in paraffin blocks. Nonetheless, we did observe correct amplification of certain old samples in the present study, likely due to suitable conditions of storage, a larger amount of DNA, or the preservation of DNA in parasite microvesicles imbedded in the paraffin.

In addition, PCR inhibitors could prevent a PCR reaction [9]. However, we controlled for the presence of inhibitors by the plasmid construction Alea in the qPCR [20] and observed no PCR inhibition.

New diagnostic methods are being evaluated for echinococcosis diagnosis, such as the detection of volatile organic compounds (VOCs) via exhaled breath analysis [39] or for CE diagnosis, via cell-free DNA (cfDNA) detection in urine [34] or blood [10]. These techniques would allow early diagnosis and population screening with a non-invasive, rapid, easy, and low-cost diagnostic tool. However, there are still problems of specificity to be overcome for the VOC method. Digital droplet PCR assays that target cfDNA in AE patient blood samples have also recently been implemented [10] and need to be tested more widely among populations at risk to validate them for diagnostic screening. Furthermore, the sensitivity can be increased by studying other DNA targets, such as tandem-repeat DNA sequences. In the same vein, high-throughput sequencing was recently used to detect cfDNA in blood samples as a non-invasive diagnostic tool [17]. However, problems of cost, implementation, and slow turnaround currently restrict their use for routine diagnosis. Nonetheless, these new, accurate, and sensitive technologies may provide new and powerful ways to obtain data from old specimens and solve the problem of handling DNA degradation, especially for retrospective studies.

**Figure 6:**
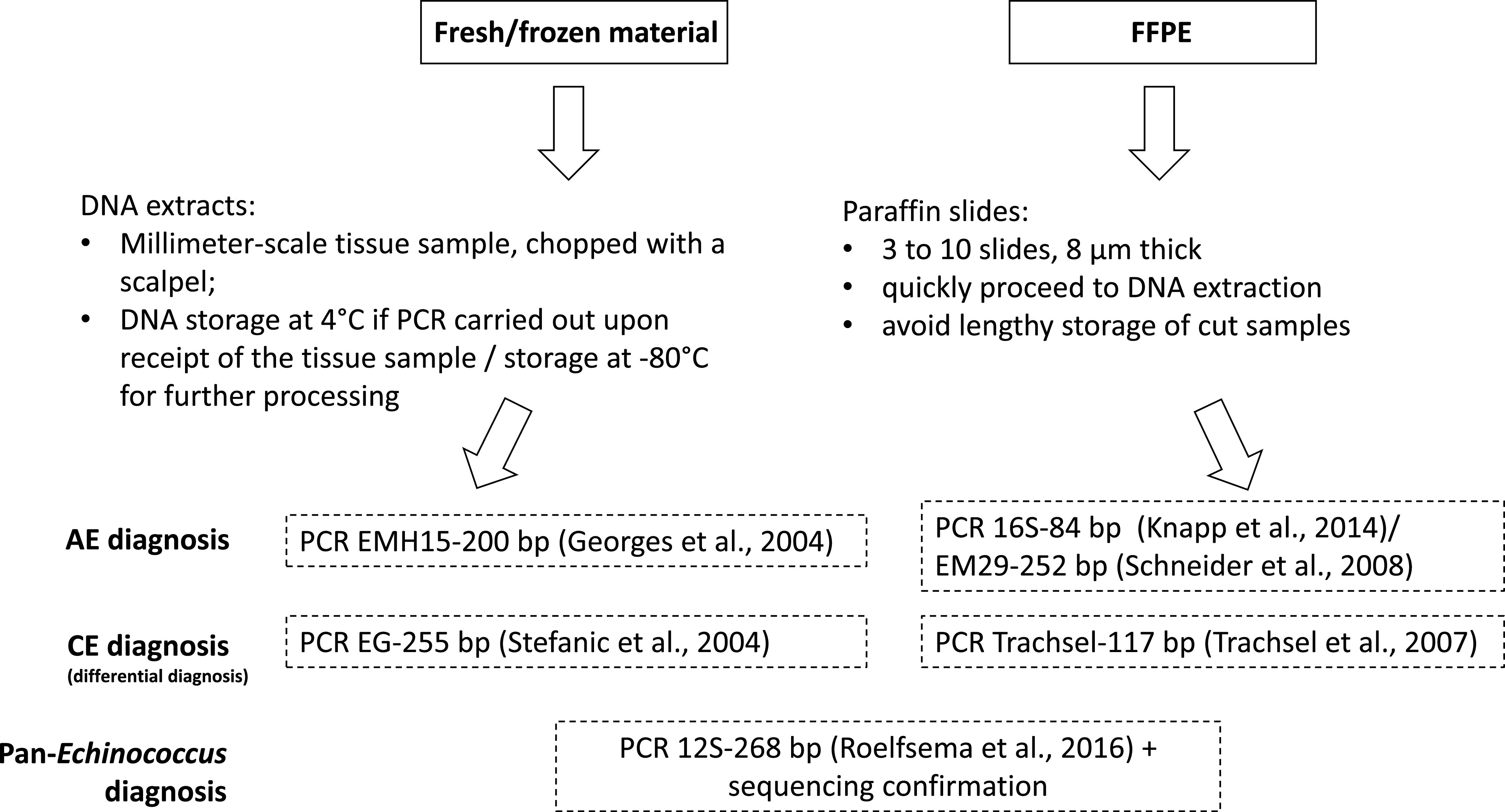
Recommendations for molecular diagnosis and differential diagnosis for alveolar echinococcosis made on suspicious *Echinococcus granulosus* isolates.

The use of molecular biology is an important element in the diagnosis of many AE cases, especially in immunosuppressed patients and for atypical localization. DNA quality can vary depending on the type of tissue conservation. We found molecular diagnosis, based on PCR, to perform better on frozen samples. For FFPE specimens, the time of storage was critical for obtaining successful PCR. Several DNA extractions using various elution volumes can be performed. Targets < 250 bp should be favored. We formulated several recommendations based on the results of the present study (Fig. 6). For initial screening of either frozen or FFPE samples, a short marker, such as the 16S-84 bp target, is recommended for use in end-point PCR or qPCR. PCR using the EMH15-200 bp or EM29-252 bp targets is advised for subsequent formal species confirmation for *E. multilocularis* identification. For differential diagnosis of *E. granulosus* isolates, the Trachsel-117 bp PCR can be chosen, especially for FFPE specimens, and the EG-255 bp PCR for fresh material. Additionally, the pan-*Echinococcus* 12S-268 bp target can be used in PCR to test for the putative occurrence of other species (e.g., *E. ortleppi* or *E. canadensis* in Europe) and/or for sequencing confirmation for non-confirmed clinical or serological echinococcosis diagnoses.

## Supplementary Materials

Supplementary material is available at https://www.parasite-journal.org/10.1051/parasite/2022004/olm

*Table S1. DNA sample panel, epidemiological and clinical details, associated to PCR results*.

## Conflict of interest

The authors declare that they have no conflict of interest.

## References

[1] Bartholomot G, Vuitton DA, Harraga S, Shi DZ, Giraudoux P, Barnish G, Wang YH, MacPherson CNL, Craig PS. 2002. Combined ultrasound and serologic screening for hepatic alveolar echinococcosis in central China. American Journal of Tropical Medicine and Hygiene, 66, 23–29. 10.4269/ajtmh.2002.66.23 12135263

[2] Baust J. 2008. Strategies for the Storage of DNA. Biopreservation and Biobanking, 6, 251–252. 24835521 10.1089/bio.2008.0604.lett

[3] Beldi G, Müller N, Gottstein B. 2017. L’échinococcose alvéolaire. Forum Médical Suisse, 17, 760–766.

[4] Bowles J, Blair D, McManus DP. 1992. Genetic variants within the genus *Echinococcus* identified by mitochondrial DNA sequencing. Molecular and Biochemical Parasitology, 54, 165–173. 1435857 10.1016/0166-6851(92)90109-w

[5] Bowles J, McManus DP. 1993. NADH dehydrogenase 1 gene sequences compared for species and strains of the genus *Echinococcus*. International Journal for Parasitology, 23, 969–972. 8106191 10.1016/0020-7519(93)90065-7

[6] Bresson-Hadni S, Bellanger A, Knapp J, Grenouillet F, Blagosklonov O, Millon L, Brumpt E, Turco C, Montange D, Richou C, Delabrousse E, Heyd B, Vuitton L, Vuitton DA. 2020. Échinococcose alvéolaire. EMC Hépatologie, 7-023-A-20.

[7] Brunetti E, Kern P, Vuitton DA, Writing Panel for the WHO-IWGE. 2010. Expert consensus for the diagnosis and treatment of cystic and alveolar echinococcosis in humans. Acta Tropica, 114, 1–16. 19931502 10.1016/j.actatropica.2009.11.001

[8] Chauchet A, Grenouillet F, Knapp J, Richou C, Delabrousse E, Dentan C, Millon L, Di Martino V, Contreras R, Deconinck E, Blagosklonov O, Vuitton DA, Bresson-Hadni S, FrancEchino Network. 2014. Increased incidence and characteristics of alveolar echinococcosis in patients with immunosuppression-associated conditions. Clinical Infectious Diseases, 59, 1095–1104. 25034426 10.1093/cid/ciu520

[9] Dietrich D, Uhl B, Sailer V, Holmes EE, Jung M, Meller S, Kristiansen G. 2013. Improved PCR performance using template DNA from formalin-fixed and paraffin-embedded tissues by overcoming PCR inhibition. PloS One, 8, e77771. 24155973 10.1371/journal.pone.0077771 PMC3796491

[10] Fan H, Gai W, Zhang L, Ma Y, Wang H, Chen X, Dong J, Zhang Y, Bao H, Zhou Y, Ren L, Cairang Y, Hou L, Ren B, Wang Z, Wang Z, Song C. 2021. Parasite circulating free DNA in the blood of alveolar echinococcosis patients as a diagnostic and treatment-status indicator. Clinical Infectious Diseases, 73, e246–e251. 33146713 10.1093/cid/ciaa1679

[11] Funabashi KS, Barcelos D, Visoná I, Silva MS e, Sousa MLAPO e, Franco MF de, Iwamura ESM. 2012. DNA extraction and molecular analysis of non-tumoral liver, spleen, and brain from autopsy samples: The effect of formalin fixation and paraffin embedding. Pathology – Research and Practice, 208, 584–591. 10.1016/j.prp.2012.07.001 22920941

[12] Georges S, Villard O, Filisetti D, Mathis A, Marcellin L, Hansmann Y, Candolfi E. 2004. Usefulness of PCR analysis for diagnosis of alveolar echinococcosis with unusual localizations: two case studies. Journal of Clinical Microbiology, 42, 5954–5956. 15583352 10.1128/JCM.42.12.5954-5956.2004 PMC535253

[13] Grimm J, Krickl J, Beck A, Nell J, Bergmann M, Tappe D, Grüner B, Barth TF, Brehm K. 2021. Establishing and evaluation of a polymerase chain reaction for the detection of *Echinococcus multilocularis* in human tissue. PLOS Neglected Tropical Diseases, 15, e0009155. 33630840 10.1371/journal.pntd.0009155 PMC7906421

[14] Guyard A, Boyez A, Pujals A, Robe C, Tran Van Nhieu J, Allory Y, Moroch J, Georges O, Fournet J-C, Zafrani E-S, Leroy K. 2017. DNA degrades during storage in formalin-fixed and paraffin-embedded tissue blocks. Virchows Archiv, 471, 491–500. 28812131 10.1007/s00428-017-2213-0

[15] Hope RM. 2013. Rmisc: Rmisc: Ryan Miscellaneous. R package version 1.5.

[16] Imrit K, Goldfischer M, Wang J, Green J, Levine J, Lombardo J, Hong T. 2006. Identification of bacteria in formalin-fixed, paraffin-embedded heart valve tissue via 16S rRNA gene nucleotide sequencing. Journal of Clinical Microbiology, 44, 2609–2611. 16825394 10.1128/JCM.00572-06 PMC1489487

[17] Ji J, Li B, Li J, Danzeng W, Li J, Zhao Y, Qiangba G, Zhang Q, Renzhen N, Basang Z, Jia C, Gongsang Q, Ma J, Wang Y, Chen F, Zhou H, Huasang, Yin J, Xie J, Pei N, Cai H, Jiang H, Yang H, Wang J, Asan, Han X, Li J, Chen W, Yang D. 2020. Comprehensive characterization of plasma cell-free *Echinococcus* spp. DNA in echinococcosis patients using ultra-high-throughput sequencing. PLoS Neglected Tropical Diseases, 14, e0008148. 32282820 10.1371/journal.pntd.0008148 PMC7209354

[18] Kern P, Bardonnet K, Renner E, Auer H, Pawlowski Z, Ammann RW, Vuitton DA, Kern P, European Echinococcosis Registry. 2003. European echinococcosis registry: human alveolar echinococcosis, Europe, 1982–2000. Emerging Infectious Diseases, 9, 343–349. 12643830 10.3201/eid0903.020341 PMC2958541

[19] Knapp J, Millon L, Mouzon L, Umhang G, Raoul F, Ali ZS, Combes B, Comte S, Gbaguidi-Haore H, Grenouillet F, Giraudoux P. 2014. Real time PCR to detect the environmental faecal contamination by *Echinococcus multilocularis* from red fox stools. Veterinary Parasitology, 201, 40–47. 24484767 10.1016/j.vetpar.2013.12.023

[20] Knapp J, Umhang G, Poulle M-L, Millon L. 2016. Development of a real-time PCR for a sensitive one-step coprodiagnosis allowing both the identification of carnivore feces and the detection of *Toxocara* spp. and *Echinococcus multilocularis*. Applied and Environmental Microbiology, 82, 2950–2958. 26969697 10.1128/AEM.03467-15 PMC4959075

[21] Koshiba M, Ogawa K, Hamazaki S, Sugiyama T, Ogawa O, Kitajima T. 1993. The effect of formalin fixation on DNA and the extraction of high-molecular-weight DNA from fixed and embedded tissues. Pathology – Research and Practice, 189, 66–72. 10.1016/S0344-0338(11)80118-4 8390645

[22] McManus DP, Gray DJ, Zhang W, Yang Y. 2012. Diagnosis, treatment, and management of echinococcosis. BMJ, 344, e3866. 22689886 10.1136/bmj.e3866

[23] Nakao M, Lavikainen A, Yanagida T, Ito A. 2013. Phylogenetic systematics of the genus *Echinococcus* (Cestoda: Taeniidae). International Journal for Parasitology, 43, 1017–1029. 23872521 10.1016/j.ijpara.2013.06.002

[24] Pawlowski ZS, Eckert J, Vuitton DA, Ammann RW, Kern P, Craig PS, Dar KF, De Rosa F, Filice C, Gottstein B, Grimm F, Macpherson CNL, Sato N, Todorov T, Uchino J, von Sinner W, Wen H. 2001. Echinococcosis in humans: clinical aspects, diagnosis and treatment, in WHO/OIE manual on echinococcosis in humans and animals: a public health problem of global concern. World Organization for Animals Health: Paris, France. p. 20–69.

[25] Piarroux M, Piarroux R, Giorgi R, Knapp J, Bardonnet K, Sudre B, Watelet J, Dumortier J, Gérard A, Beytout J, Abergel A, Mantion G, Vuitton DA, Bresson-Hadni S. 2011. Clinical features and evolution of alveolar echinococcosis in France from 1982 to 2007: results of a survey in 387 patients. Journal of Hepatology, 55, 1025–1033. 21354448 10.1016/j.jhep.2011.02.018

[26] R Development Core Team. 2018. R Development Core Team (2018). R: A language and environment for statistical computing. R Foundation for Statistical Computing: Vienna, Austria. http://www.R-project.org.

[27] Reuter S, Jensen B, Buttenschoen K, Kratzer W, Kern P. 2000. Benzimidazoles in the treatment of alveolar echinococcosis: a comparative study and review of the literature. Journal of Antimicrobial Chemotherapy, 46, 451–456. 10.1093/jac/46.3.451 10980173

[28] Roelfsema JH, Nozari N, Pinelli E, Kortbeek LM. 2016. Novel PCRs for differential diagnosis of cestodes. Experimental Parasitology, 161, 20–26. 26704662 10.1016/j.exppara.2015.12.010

[29] Romig T, Deplazes P, Jenkins D, Giraudoux P, Massolo A, Craig PS, Wassermann M, Takahashi K, de la Rue M. 2017. Ecology and life cycle patterns of *Echinococcus* species. Advances in Parasitology, 95, 213–314. 28131364 10.1016/bs.apar.2016.11.002

[30] Schneider R, Gollackner B, Edel B, Schmid K, Wrba F, Tucek G, Walochnik J, Auer H. 2008. Development of a new PCR protocol for the detection of species and genotypes (strains) of *Echinococcus* in formalin-fixed, paraffin-embedded tissues. International Journal for Parasitology, 38, 1065–1071. 18177654 10.1016/j.ijpara.2007.11.008

[31] Stefanić S, Shaikenov BS, Deplazes P, Dinkel A, Torgerson PR, Mathis A. 2004. Polymerase chain reaction for detection of patent infections of *Echinococcus granulosus* (“sheep strain”) in naturally infected dogs. Parasitology Research, 92, 347–351. 14727186 10.1007/s00436-003-1043-y

[32] Thompson RCA. 2020. The molecular epidemiology of *Echinococcus* Infections. Pathogens, 9, E453. 32521787 10.3390/pathogens9060453 PMC7350326

[33] Torgerson PR, Keller K, Magnotta M, Ragland N. 2010. The global burden of alveolar echinococcosis. PLoS Neglected Tropical Diseases, 4, e722. 20582310 10.1371/journal.pntd.0000722 PMC2889826

[34] Toribio L, Santivanez S, Scott AL, Enriquez R, Sedano C, Soto-Becerra P, Garcia HH, Shiff CJ, Cysticercosis Working Group in Peru. 2020. Diagnostic urinary cfDNA detected in human cystic echinococcosis. Molecular and Biochemical Parasitology, 239, 111314. 32866606 10.1016/j.molbiopara.2020.111314 PMC8801304

[35] Trachsel D, Deplazes P, Mathis A. 2007. Identification of taeniid eggs in the faeces from carnivores based on multiplex PCR using targets in mitochondrial DNA. Parasitology, 134, 911–920. 17288631 10.1017/S0031182007002235

[36] Vuitton DA, Zhou H, Bresson-Hadni S, Wang Q, Piarroux M, Raoul F, Giraudoux P. 2003. Epidemiology of alveolar echinococcosis with particular reference to China and Europe. Parasitology, 127(Suppl), S87–107. 15027607

[37] Vuitton DA, Bresson-Hadni S. 2014. Alveolar echinococcosis: evaluation of therapeutic strategies. Expert Opinion on Orphan Drugs, 2, 67–86.

[38] Vuitton DA, McManus DP, Rogan MT, Romig T, Gottstein B, Naidich A, Tuxun T, Wen H, Menezes da Silva A, World Association of Echinococcosis. 2020. International consensus on terminology to be used in the field of echinococcoses. Parasite, 27, 41. 32500855 10.1051/parasite/2020024 PMC7273836

[39] Welearegay TG, Diouani MF, Österlund L, Borys S, Khaled S, Smadhi H, Ionescu F, Bouchekoua M, Aloui D, Laouini D, Cindemir U, Ionescu R. 2019. Diagnosis of human echinococcosis via exhaled breath analysis: a promise for rapid diagnosis of infectious diseases caused by helminths. Journal of Infectious Diseases, 219, 101–109. 10.1093/infdis/jiy449 30016445

[40] Wen H, Vuitton L, Tuxun T, Li J, Vuitton DA, Zhang W, McManus DP. 2019. Echinococcosis: advances in the 21st century. Clinical Microbiology Reviews, 32, e00075–e00018. 30760475 10.1128/CMR.00075-18 PMC6431127

[41] Zhang W, McManus DP. 2006. Recent advances in the immunology and diagnosis of echinococcosis. FEMS Immunology and Medical Microbiology, 47, 24–41. 16706785 10.1111/j.1574-695X.2006.00060.x

